# Draft Genome Sequence of *Comamonas testosteroni* R2, Consisting of Aromatic Compound Degradation Genes for Phenol Hydroxylase

**DOI:** 10.1128/genomeA.00875-17

**Published:** 2017-09-07

**Authors:** Fatma Azwani, Kenshi Suzuki, Masahiro Honjyo, Yosuke Tashiro, Hiroyuki Futamata

**Affiliations:** a Institute of Tropical Agriculture and Food Security, Universiti Putra Malaysia, Serdang, Selangor, Malaysia; b Department of Applied Chemistry and Biochemical Engineering, Graduate School of Engineering, Shizuoka University, Hamamatsu, Shizuoka, Japan; c Graduate School of Science and Technology, Shizuoka University, Hamamatsu, Shizuoka, Japan; d Research Institute of Green Science and Technology, Shizuoka University, Suruga-ku, Shizuoka, Japan

## Abstract

*Comamonas testosteroni* strain R2 was isolated from a continuous culture enriched by a low concentration of phenol-oxygenating activities with low *K*_*s*_ values (below 1 μM). The draft genome sequence of *C. testosteroni* strain R2 reported here may contribute to determining the phenol degradation gene cluster.

## GENOME ANNOUNCEMENT

The genome sequencing of many environmental microbes, such as *Comamonas* spp. (1) and *Pseudomonas* spp. (2), has been carried out to better understand the ability of these organisms to use aromatic compounds as sources of carbon. The genome data for these organisms will contribute to our understanding of interspecies interactions and microbial community dynamics (3) and also provide significant insight to the development of bioremediation technologies. Our previous study showed that *C. testosteroni* R2, which was isolated from a chemostat, expressed phenol-oxygenating activities with low apparent *K*_*s*_ (below 1 μM) and had the ability to utilize phenol as a carbon source (4–6).

In order to predict the cluster involved in the expression of the phenol hydroxylase gene of *C. testosteroni* R2, a draft genome sequence was run. The genomic DNA of strain R2 was extracted using a commercial DNA isolation kit, and genome sequencing was performed using a combined method of whole-genome shotgun and paired-end sequencing (7, 8). Draft genome sequence data for strain R2 were generated using 454 GS-FLX Titanium paired-end data (Roche, Basel, Switzerland) (8), which consisted of 631,574 chemistry reads and a total of 189,459,207 bp of sequencing data. The removal of adapter sequences and quality trimming were performed in all data sets prior to *de novo* assembly to correct potential base errors and increase consensus quality. Newbler GS *de novo* assembler version 2.5 software (8) was used to assemble the reads into five scaffolds, with an *N*_50_ length of 34,745 bp. The draft genome sequence of R2 was estimated to comprise 5,871,018 bp.

The resulting DNA scaffolds, as translational products of coding sequences, were further analyzed by searching the GenBank database to find predicted protein-coding genes, tRNAs, and rRNAs. By using a combination of the Rapid Annotations using Subsystems Technology (RAST) server (9) and the Microbial Genome Annotation Pipeline (MiGAP) (http://www.migap.org), the genome was estimated to have an overall G+C content of 60.9%. In total, 5,512 coding regions and 61 tRNAs were predicted and annotated. The rRNAs were further identified using the Southern blot hybridization protocol, which contains five 5S-16S-23S clusters.

The genes encoding phenol hydroxylase of strain R2 were identified. This strain contained one multicomponent phenol hydroxylase and one phenol-metabolic pathway. Under aerobic conditions, phenol hydroxylase is responsible for converting phenol to catechol (10) by incorporating a single hydroxyl group into the substrate—the initial and rate-limiting step in phenol degradation pathways (11). This hydroxylation is followed by ring cleavage that converts catechol by catechol 2,3-dioxygenase C23O (12) to the other metapathway enzymes, such as pyruvate, succinate, and acetyl coenzyme A. Information about the genome sequence of *C. testosteroni* R2 will be helpful for understanding the diversity and mechanisms of phenol degradation in the environment and for furthering bioremediation research.

### Accession number(s).

The draft genome sequence of strain R2 has been deposited in the DDBJ/EMBL/GenBank database under the accession no. BDQJ01000001 to BDQJ01000005.
